# The Effect of Metabolic Control on Diabetes Complication Rates and the Need for Medical Care During COVID-19 Social Isolation in Adjara, Georgia

**DOI:** 10.7759/cureus.51093

**Published:** 2023-12-25

**Authors:** Liana Jashi, Rusudan Kvanchakhadze, Tamar Peshkova, Ketevan Dundua, Lela Nakaidze, Eter Margalitadze, Tebrone Gachechiladze

**Affiliations:** 1 Medicine, David Agmashenebeli University of Georgia, Tbilisi, GEO; 2 Medicine, Avicenna – Batumi Medical University, Batumi, GEO; 3 Endocrinology, National Center for Disease Control and Public Health (NCDC), Tbilisi, GEO; 4 Medicine, Batumi Shota Rustaveli State University, Batumi, GEO; 5 Medicine, David Aghmashenebeli University of Georgia, Tbilisi, GEO

**Keywords:** complications, metabolic control, diabetes mellitus, covid-19, social isolation

## Abstract

Introduction: Social isolation or distancing during the COVID-19 pandemic was associated with poor metabolic control in patients with diabetes mellitus. It might have contributed to the high mortality rate of those with diabetes who became infected. This study aims to determine the degree or level of metabolic control in patients with diabetes mellitus, the progression of its complications, and the need for emergency care during social isolation caused by the COVID-19 pandemic.

Materials and methods: A retrospective study was conducted in Georgia on 752 diabetic patients aged under 65 years old. Results showed that many patients did not control their blood glucose levels, measure their blood pressure, or know their cholesterol and glycated hemoglobin levels before and after the pandemic. Over 35% of patients experienced glycemic profile fluctuations. We compared metabolic rates with complications of diabetes and the need for emergency medical care during isolation. It was found that the testability of glycemia (p = 0.006), fluctuations in glucose (p = 0.001), and glycated hemoglobin levels before (p = 0.001) and after the pandemic (p = 0.004) increased the prevalence of diabetes-related heart disease and multiple micro- and macroangiopathies. Blood pressure (p = 0.001), cholesterol levels (p = 0.001), and glucose control (p = 0.012) affected the condition of patients with diabetes mellitus. It increased the need for medical care due to infarction, hypertension crisis, and hyperglycemia.

Conclusions: In a crisis where medical care is limited, the management of diabetes patients requires more attention. Our study proves that active middle-aged patients during isolation had poor metabolic control in terms of self-control of the disease. It is necessary to constantly inform and educate patients about the importance of metabolic parameters in progressing diabetes complications. Proper metabolic control could prevent complications of diabetes and improve a patient's quality of life.

## Introduction

On March 11, 2020, the WHO declared COVID-19, the viral disease, a pandemic, currently considered the greatest challenge the world has ever faced [1]. The virus rapidly spreading and causing disease has significantly impacted people's lives and personal relationships. Due to the COVID-19 pandemic posing a serious threat, social distancing and isolation have been recognized as reliable and effective measures to mitigate the spread of the virus, which has been well demonstrated in China [2]. Since these measures lasted over time, short-term and long-term secondary damages amidst social isolation should be considered when assessing the risk. Older adults, people with underlying medical conditions, and immunocompromised individuals are at a higher risk of severe illness [3].

In the COVID-19 pandemic era, diabetes was a major risk factor for fatal COVID-19 outcomes [4]. The CONFI-DIAB study analyzed the management of diabetes during the COVID-19 lockdown period, focusing on glucose control, weight changes, healthcare utilization, and the use of telemedicine services. They analyzed data from 870 patients with diabetes, finding a significant reduction in glycated hemoglobin (HbA1c) levels despite the lockdown and disruption in healthcare. Stratified analyses indicated a consistent and significant reduction of HbA1c levels, regardless of diabetes type. Weight changes significantly affected HbA1c reduction. There was no deterioration, but rather an improvement in metabolic control [5]. During the COVID-19 lockdown in India, previously treated type 2 diabetes mellitus (T2DM) patients were studied. In telephonic interviews with 150 patients, 21% increased carbohydrate intake, 23% snacked more, and 27% ate more fruits.

The lockdown may destabilize or exacerbate T2DM, but positive changes were also observed [6]. It should be emphasized that people's lifestyles have changed significantly across the globe, including Georgia. In this context, the prevalence of multiple chronic conditions has increased. Moreover, along with transport restrictions and the economic crisis, a significant portion of the population has encountered barriers to accessing crucial medical services [7]. Numerous global studies have been carried out to examine the effects of social isolation on mental health, mainly focusing on the rise in feelings of loneliness [8]. There are many literature reviews of the studies in this regard, clarifying the effect of isolation on changes in people's mental health. According to these studies, from the very beginning, there was evidence that the mortality rate in patients with diabetes due to COVID-19 was significantly higher compared to other patients. In particular, the mortality rate was 7.8% and 2.7% in China, 28.8% and 6.2% in the USA, 31.4% and 14.4% in England, and 15.2% in Russia [9,10]. During the COVID-19 lockdown in Turkey, they studied the impact on 101 T2DM patients who could not attend regular follow-ups. They found that patients’ mean weight increased, but it was not statistically significant. Similarly, they observed non-significant increases in HbA1c and fasting glucose levels. However, this study suggests lockdowns may not significantly impact glycemic parameters in T2DM patients [11]. In Italy, during the lockdown imposed on March 9th, 2020, due to COVID-19, a study analyzed the continuous glucose monitoring (CGM) data of 55 adults with diabetes, selecting those who were able to upload data and hadn't had contact with diabetologists for therapeutic changes. The data was analyzed 14 days before and after the lockdown. The analysis showed that the lockdown resulted in lifestyle changes that affected glucose control, particularly for those on complex therapeutic regimens [12].

Metabolic control of patients with diabetes mellitus varies around the world and depends on the strictness of isolation measures and access to healthcare services [13,14]. A deterioration of metabolic parameters in patients with diabetes mellitus was observed in countries with low socioeconomic development [15,16], while in highly developed countries, there tended to be an improvement. The study conducted on 63 patients with diabetes in Italy amidst isolation reported improvements in metabolic parameters [17], while a deterioration in the same parameters was observed in India. In Brazil, an initial deterioration was observed after analyzing 24-month data, especially among the patients receiving public services [18]. The Nordic study group published another extensive review describing the frequency of loneliness being experienced and the emotional status of the patients [19].

The first case of a COVID-19 patient in Georgia was reported in February 2020. Following this, the country had to undergo multiple shutdowns to contain the spread of the virus. The pandemic hit Georgia in four waves, causing high mortality rates. As of October 2021, the number of deaths due to COVID-19 was 8976, resulting in a mortality rate of 1.46%. The mortality rate was highest among older patients, with 70- to 74-year-olds having a mortality rate of 12.8% and 80- to 84-year-olds having a rate of 18.05%. Middle-aged people were not immune to the virus, with 55- to 59-year-olds having a mortality rate of 6.78% and 65- to 69-year-olds having a rate of 14.5%. Since April 2020, Tbilisi has had the highest mortality rate due to COVID-19. The exception was from October to November 2020, with the highest mortality rate reported in the Ajara region [20]. Researchers mostly focus on the elderly population with some chronic diseases; few data are available in the <65 age group. Therefore, this study aimed to determine the level of metabolic control, the progression of complications, and the need for outpatient or emergency care in middle-aged patients with diabetes during the period of social isolation caused by the pandemic.

## Materials and methods

This is the first study conducted in Georgia among patients with diabetes under 65 years old during the social isolation implemented during the COVID-19 pandemic. While the pandemic has slowed down, the prevalence of diabetes and the prevalence of associated complications are increasing worldwide, especially among relatively young people of working age. As part of our research, we utilized a retrospective analytical cross-sectional design and a specialized survey questionnaire. We followed the WHO STEPwise approach to surveillance (STEPS) of chronic disease risk factors, and our research team developed questionnaires. These questionnaires were adapted for patients with diabetes mellitus based on the study's specific goals. The questionnaires were moved to an online platform using Google Forms (Alphabet Inc., Mountain View, CA, USA). A pilot study was conducted to ensure the questionnaires' validity and reliability, resulting in a Cronbach’s alpha coefficient of 0.73 for patient questionnaires. According to the standards set by George and Mallery (2003) [21], this value is considered acceptable and valid. These standards require scores above 0.7.

We used inclusion and exclusion criteria for the patient population to determine our sample size. Individuals between the ages of 20 and 65 who had been living with diabetes for more than a year were included in the study. Patients who were not diabetic, those younger than 20 years old or older than 65, those who had been diagnosed with diabetes for less than a year, and those who refused to participate were excluded from the study. Based on a confidence level of 95%, an acceptable margin of error of 5%, and the prevalence of diabetes in Georgia (6.8%), we determined a primary sample size of 99 people. However, considering the "design effect," the sample size required for data analysis was 594 respondents. With a response rate of 80%, the number of respondents with diabetes mellitus was 742.

To collect information, we used a combination of online surveys, face-to-face interviews, and visits to various regions. Statistical data analysis was performed using SPSS Statistics version 27 (IBM Corp., Armonk, NY, USA). Descriptive statistics are presented as mean absolute numbers and percentages. We used the chi-square value to analyze such differences in categorical variables and test whether the variables were related. A p-value <0.05 was used as a 95% confidence interval cut-off point for all statistical significance.

Our questionnaire included sociodemographic characteristics, metabolic control, current treatment and barriers, changes in habits, emotional state, need for emergency care, and hospitalization. In this article, we look at the impact of changes in metabolic measures (glycemic testability, glycemic variability, blood pressure, and cholesterol control) on the progression of complications in patients and the need for outpatient or emergency care in social isolation settings.

Barriers to the study

Refusal to participate or partially completed questionnaires were considered barriers. The study started in March 2022 and was continued in February 2023. Endocrinologists from five clinics contacted patients. The time for filling out the online survey was about 15 to 20 minutes. We also visited high-mountain regions in Ajara. Written, informed consent was obtained from each participant. Georgia does not have an electronic registration system, making patient contact difficult. We had no previous information on patients' metabolic control or existing complications. The information received was provided directly by the patients.

## Results

A total of 752 patients were involved in the study, of whom 725 agreed to complete the survey questionnaire. Twenty-seven respectfully refused. The sociodemographic characteristics and distribution of patients according to gender, nationality, age, and social status are presented in Table 1.

**Table 1:** Sociodemographic characteristics of patients LADA: Latent autoimmune diabetes of adults, MODY: Maturity-onset diabetes of the young

Variables	Characteristics	n	%
Gender	Female	392	55.2
	Male	318	44.8
Where do you live?	Batumi	314	44.2
	Kobuleti	165	23.2
	Khelvachauri	139	19.6
	Highland Ajara	92	13
Religion	Christian	505	72.1
	Muslim	175	25
	Not Religious	20	2.9
Age	20-30	10	1.4
	31-40	32	4.4
	41-50	168	23.3
	51-60	354	49.2
	60-65	156	21.7
Marital status	Married	412	58
	Divorced	89	12.5
	Never married	78	11
	Widow	131	18.5
Education	Incomplete secondary	50	7.1
	Secondary	101	14.2
	Secondary technical education	194	27.4
	Incomplete higher	131	18.5
	Higher	229	32.3
	Scientific degree	4	0.6
Working Status	Employed	359	50.6
	Unemployed	179	25.2
	Lost his/her job due to the pandemic	106	15
	Pensioner	65	9.2
Insurance	Universal (state) insurance	486	68.6
	Private insurance	161	22.7
	Do not have Insurance	61	8.6
Diabetes medical history	1-3 years	151	21.3
	3-5 years	17	2.4
	5-10 years	236	33.2
	More than 10 years	306	43.1
What type of diabetes do you have?	Type 1	31	4.4
	Type 2	617	87.3
	LADA	52	7.4
	MODY	7	1

According to the control of the main metabolic indicators (glucose (29%), HbA1c before isolation (63.8%) and after (59.4%), blood pressure (22.7%), and cholesterol (37.1%)), many patients did not undergo testing. As for fluctuations in the glycemic profile, most patients had fluctuations in glucose levels in the range of 140 to 250 mg/dL and more than 250 mg/dL more (35.7% vs. 29.3%). The glycated hemoglobin level before isolation was predominantly higher than 9% (10.9%) and 7% to 8% (12.4%), while only 5.6% reported having a level of 6% to 7%. After isolation, most patients scored lower (18.9%) or higher (15.4%) than 8%. During isolation, the majority of respondents (40.3%) had an increase in blood pressure in the range of 140/90 mmHg, and only 12% of patients needed medical assistance due to an increase in blood pressure of 180 mmHg or more. Most patients also had high total cholesterol (36.6%) and were on medication. The most common complications were multiple micro-macroangiopathies (retinopathies, nephropathies, neuropathies, cardiovascular diseases, cerebral vascular diseases, and diabetic foot). Two or more complications (29.3%) and arterial hypertension crisis (34.5%) were the main reasons for seeking outpatient treatment, and heart attacks (18.7%) were the reason for seeking inpatient treatment. The descriptive statistics for the questionnaire factors are presented in Table 2. 

**Table 2:** Descriptive statistics of questionnaire factors DPP-4: Dipeptidyl peptidase 4, SGLT-2: Sodium-glucose cotransporter-2, GLP-1: Glucagon-like peptide 1, HbA1c: Glycated hemoglobin

Factors	Response	n	%
Medical treatment/therapy that you are taking	Insulin therapy with insulin analogs	121	16.8
	Insulin therapy, the combination of Actrapid and Insultard	173	24
	Metformin, sulphonylurea, combination	210	29
	DPP-4 inhibitors	139	19
	SGLT-2 inhibitors	59	8.2
	GLP-1 agonists	19	2.6
How often do you check your blood sugar?	I don't check	209	29.6
	Once a month	1	0.1
	Once a week	231	32.7
	Once a day	113	16
	Several times a day	153	21.6
Blood sugar level fluctuation while in isolation	Periodically it was low, less than 100mg/dl	101	15.3
	It was normal, from 90mg/dl to 120mg/dl	130	19.7
	Fluctuated within 140 mg/dl to 250 mg/dl	236	35.7
	Systematically, it was more than 250 mg/dl	194	29.3
HbA1c levels before the pandemic	6% to 7%	40	5.6
	7% to 8%	77	10.9
	8% to 9%	52	7.3
	Over 9%	88	12.4
	Don’t know	452	63.8
HbA1c levels after pandemic-related isolation	Increased by more than 8%	109	15.4
	More than 9%	25	3.5
	Decreased, less than 8%	134	18.9
	Decreased, less than 7%	20	2.8
	Don’t know	421	59.4
Your blood pressure during the isolation	Usually, I have low blood pressure; 100/70mmHg	88	12.4
	It was normal; 120/80mmHg	88	12.4
	Elevated at 140/90mmHg	286	40.3
	Seeking medical care due to the high BP; 180mmHg or higher	86	12.1
	I have not checked it	161	22.7
What was your cholesterol level?	In norm	74	10.5
	It is high; not taking medicine	111	15.8
	It was high; taking medicine	258	36.6
	Don't know	261	37.1
Complications of diabetes	Cardiological diseases of diabetes	133	19.1
	Multiple macroangiopathy (cardiovascular disease, diabetic foot, cerebrovascular disease)	104	14.9
	Multiple microangiopathy (diabetic retinopathy, diabetic nephropathy, two or more diabetic neuropathy together)	151	21.7
	Multiple micro- and macroangiopathy	204	29.3
	No	104	14.9
Did you need help while in isolation or quarantine?	Outpatient care due to hypoglycemia	53	9.2
	Outpatient care due to high blood pressure	200	34.5
	Other diseases/surgical intervention	41	7.1
	Inpatient care due to heart attack	108	18.7
	Inpatient care due to hyperglycemia	58	10
	No	119	20.6

During the study, metabolic parameters were analyzed in relation to the prevalence of diabetes complications and the need for assistance during isolation. The data showed that controlling glucose levels was crucial in preventing diabetes-related complications. Patients who controlled their glucose levels at least once a week had a lower prevalence of complications (40%) than those who did not control their glucose levels (17%) at all and had a higher prevalence of multiple micro and macrovascular diseases (36.82%), multiple microangiopathies (31.51%), multiple macrovascular diseases (23%), and diabetic-related cardiological diseases (28%). The chi-square value was 33.42 with a p-value of 0.006, indicating a significant association between glucose control and diabetes complications.

The prevalence of multiple micro- and macroangiopathy in patients who constantly had 250 mg% or more was 41.49%, compared to 35.11% in patients with fluctuations in the 140 to 250 mg% range. The prevalence of cardiovascular diseases in diabetes mellitus was high, with glucose fluctuations in the range of 140 to 250 mg% (43.22%) and 250 mg% or more (22.8%). Patients in the 140 to 250 mg% group had a higher prevalence of multiple microangiopathy (33.09%) than those who had a consistently high rate (27.95%). The chi-square value was 40.83, and the p-value was 0.001.

Before the pandemic, many patients did not know their glycated hemoglobin levels. Of those who were not tested, 62.78% had cardiac problems, 71.29% had multiple macroangiopathies, 56.61% had multiple microangiopathies, and 69.15% had multiple micro- and macroangiopathies. The most common multiple microangiopathy was observed in the group with glycated hemoglobin above 9% (18.24%), whereas the prevalence of diabetes mellitus, cardiological diseases, and multiple micro- and macroangiopathies was highest in the 7% to 8% group (13.18% and 11.94%, respectively). The prevalence of heart disease was highest in the 6% to 7% group (9.3%). The chi-square value was 39.85, and the p-value was 0.001.

However, the glycated hemoglobin level after isolation in the group is unknown. In this group, multiple macroangiopathy was seen in 66.67%, and multiple micro- and macroangiopathy in 67%. The frequency of heart disease was also quite high, at 58.14%. With glycosylation above 8%, there was an increase in heart diseases by 20.16% and multiple microangiopathies by 18%. On the other hand, a decrease in glycated hemoglobin below 8% decreased cardiovascular disease by 17.05%. However, coronary macroangiopathy increased by 19.61% and microangiopathy by 24.67%. This was indicated by a chi-square value of 34.86 and a p-value of 0.004.

Multiple micro- and macroangiopathies increased by 27.54% when blood pressure was not checked. With an increase in blood pressure above 140/90mmHg, the growth of all complications was almost the same, and the prevalence of cardiovascular diseases due to diabetes mellitus was 43.94%. The chi-square value was 23.86, and the p-value was 0.093. Cholesterol levels increase the prevalence of diabetes complications. Patients who did not take the drug had a 15.91% increase in the prevalence of cardiovascular diseases related to diabetes, whereas those who took the drug had a 42.42% increase. The chi-square value was 14.94, and the p-value was 0.245. A cross-tabulation to assess factors associated with complications of diabetes is presented in Table 3.

**Table 3:** A cross-tabulation to assess factors associated with complications of diabetes ^1^multiple microangiopathies (retinopathy, nephropathy, neuropathy) ^2^multiple macroangiopathies (cardiovascular disease, cerebrovascular disease, diabetic foot) ^3^multiple micro- and macroangiopathies (retinopathy, nephropathy, neuropathy, cardiovascular disease, cerebrovascular disease, diabetic foot, two or more from this)

Factors		Cardiological diseases of diabetes		Multiple macroangiopathy^1^		Multiple microangiopathy^2^		No		Multiple micro-macroangiopathy^3^		chi-square value	p-value
		n	%	n	%	n	%	n	%	n	%		
How often do you check your blood sugar?	Once a week	34	25.76	44	42.31	45	30.82	40	40.82	60	29.85	33.42	0.006
	I didn't check	37	28.03	24	23.08	46	31.51	17	17.35	74	36.82		
	Several times a day	37	28.03	20	19.23	28	19.18	29	29.59	35	17.41		
	Once a day	23	17.42	15	14.42	23	15.75	11	11.22	32	15.92		
	Once a month	1	0.76	1	0.96	4	2.74	1	1.02	0	0		
Blood sugar level fluctuation while in isolation	Periodically, it was low, less than 100mg/dl	15	12.71	14	14.43	24	17.65	22	21.15	24	12.77	40.83	
	Systematically it was more than 250mg/dl	27	22.88	33	34.02	38	27.94	17	16.35	78	41.49		0.001
	Fluctuated within 140mg/dl - 250mg/dl	51	43.22	33	34.02	45	33.09	32	30.77	66	35.11		
	It was in the norm, from 90mg/dl to 120mg/dl	25	21.19	17	17.53	29	21.32	33	31.73	20	10.64		
Glycated hemoglobin levels before the pandemic	6% to 7%	12	9.3	2	1.98	11	7.43	4	3.88	10	4.98	39.85	0.001
	Don’t know	81	62.79	72	71.29	84	56.76	50	48.54	139	69.15		
	8% to 9%	7	5.43	11	10.89	14	9.46	9	8.74	11	5.47		
	Over 9%	12	9.3	9	8.91	27	18.24	23	22.33	17	8.46		
	7% to 8%	17	13.18	7	6.93	12	8.11	17	16.5	24	11.94		
Glycated hemoglobin levels after pandemic-related isolation	Increased by more than 8%	26	20.16	9	8.82	27	18	16	15.84	28	14	34.86	0.004
	Don’t know	75	58.14	68	66.67	75	50	45	44.55	134	67		
	More than 9%	2	1.55	5	4.9	7	4.67	5	4.95	6	3		
	Decreased, less than 8%	22	17.05	20	19.61	37	24.67	29	28.71	26	13		
	Decreased, less than 7%	4	3.1	0	0	4	2.67	6	5.94	6	3		
Your blood pressure during the isolation	In the norm - 120/80mm/Hg	23	17.42	6	5.83	24	16.55	12	12	22	10.84	23.86	0.093
	Elevated - 140 /90mmHg	58	43.94	44	42.72	58	40	43	43	75	36.95		
	Seeking medical care due to high BP- 180mmHg or higher	21	15.91	16	15.53	12	8.28	9	9	23	11.33		
	I have not checked it	18	13.64	23	22.33	32	22.07	25	25	56	27.59		
	Usually, I have low blood pressure - 100/70mmHg	12	9.09	14	13.59	19	13.1	11	11	27	13.3		
What was your cholesterol level?	Don't know	46	34.85	33	32.67	55	37.67	38	38	81	40.7	14.94	0.245
	It was high; not taking medicine	21	15.91	12	11.88	24	16.44	12	12	35	17.59		
	It was high; taking medicine	56	42.42	41	40.59	51	34.93	44	44	60	30.15		
	In the norm	9	6.82	15	14.85	16	10.96	6	6	23	11.56		

The study also found that patients who did not control their glucose levels had a higher need for assistance during isolation. Around 40.19% of them needed help due to heart attacks, 31.58% due to hyperglycemia, 36.59% due to other diseases or surgery, and 32% due to a high blood pressure crisis. Patients who controlled their glucose levels every day had a lower need for assistance (28%) compared to those who did not control their glucose levels at all. However, the daily control group had a higher prevalence of high blood pressure (35.85%) and hypoglycemia (17%). Patients who controlled their glucose levels once a week had the highest frequency of hyperglycemia (38.6%) and infarction (33%). The chi-square value was 37.04 with a p-value of 0.012, indicating a significant association between glucose control and the need for assistance during isolation.

However, hospitalizations for hyperglycemia were highest in patients with glucose levels ranging from 140 to 250 mg% (46.15%), followed by infarction (32.29%) and hypertension crisis (37.16%). For glucose levels above 250 mg%, outpatient care was required due to an arterial hypertensive crisis (26.68%) and an infarction (29.17%). The chi-square value was 15.92, and the p-value was 0.387.

After isolating patients unaware of their glycosylated hemoglobin levels, it was found that the need for assistance was still high. For patients whose levels were above 8%, there were high rates of arterial crisis (17.86%), hyperglycemia (21.05%), and infarction (16.19%). The chi-square value is 20.36, and the p-value is 0.435.

In the absence of measurement of blood pressure, the need for help was high due to hypoglycemia (32.08%), hyperglycemia (26.32%), and heart attack (29.63%). In patients with a blood pressure of 180mmHg or more, the need for help was high due to a crisis (24.62%) and infarctions (23.15%). Patients with an increased blood pressure of 140/90 mmHg had a 54.77% risk of crisis, a 31.48% risk of heart attack, a 33.96% risk of hypoglycemia, and a 36.84% risk of hyperglycemia. The chi-square value was 136.29, and the p-value was 0.001.

Those with high cholesterol and who used medication were more likely to need outpatient care for a high blood pressure crisis and inpatient care in the event of a heart attack than patients who had high cholesterol but did not take medication: 41.3% vs. 24.4% and 51.4% vs. 15.8%. The distribution is plausible: chi-square value = 48.34, p-value = 0.001. A cross-tabulation to assess factors associated with the need for emergency care is presented in Table 4.

**Table 4:** A cross-tabulation to assess factors associated with the need for emergency care

		Did you need help while in isolation/quarantine?													
Factors		Outpatient care, due to hypoglycemia		Outpatient care due to high blood pressure		Other diseases / surgical intervention		No		Inpatient care due to heart attack		Inpatient care due to hyperglycemia		Chi2 value	p-value
		n	%	n	%	n	%	n	%	n	%	n	%		
How often do you check your blood sugar?	Once a week	17	32.08	66	33	13	31.71	37	31.62	36	33.64	22	38.6	37.04	0.012
	I didn't check	10	18.87	64	32	15	36.59	22	18.8	43	40.19	18	31.58		
	Several times a day	19	35.85	35	17.5	5	12.2	33	28.21	12	11.21	10	17.5		
	Once a day	7	13.21	34	17	8	19.51	22	18.8	14	13.08	7	12.28		
	Once a month	0	0	1	0.5	0	0	3	2.56	2	1.87	0	0		
Blood sugar level fluctuation while in isolation	Periodically, it was low, less than 100mg/dl	6	12	35	19.13	2	5	14	12.73	16	16.67	7	13.46	15.92	0.387
	Systematically it was more than 250mg/dl	19	38	49	26.78	15	37.5	31	28.18	28	29.17	10	19.23		
	Fluctuated within 140 mg/dl - 250mg/dl	13	26	68	37.16	14	35	38	34.55	31	32.29	24	46.15		
	It was in the norm, from 90mg/dl to 120mg/dl	12	24	31	16.94	9	22.5	27	24.55	21	21.88	11	21.15		
Glycated hemoglobin levels before the pandemic	6% to 7%	6	11.54	12	6.03	2	5.13	4	3.51	7	6.6	4	6.9		0.435
	Don’t know	35	67.31	110	55.28	27	69.23	77	67.54	70	66.04	39	67.24		
	8% to 9%	4	7.69	19	9.55	4	10.26	7	6.14	4	3.77	4	6.9		
	Over 9%	2	3.85	34	17.09	3	7.69	13	11.4	13	12.26	4	6.9		
	7% to 8%	5	9.6	24	12.06	3	7.69	13	11.4	12	11.32	7	12.07		
Glycated hemoglobin levels after pandemic-related isolation	Increased, by more than 8%	9	16.98	35	17.86	5	12.5	15	12.93	17	16.19	12	21.05	26.55,	
	Don’t know	32	60.38	102	52.04	25	62.5	75	64.66	64	60.95	35	61.4		0.148
	More than 9%	4	7.55	3	1.53	2	5	2	1.72	4	3.81	1	1.75		
	Decreased, less than 8%	6	11.32	54	27.55	6	15	20	17.24	17	16.19	7	12.28		
	Decreased, less than 7%	2	3.77	2	1.02	2	5	4	3.45	3	2.86	2	3.51		
Your blood pressure during the isolation	In norm - 120/80mmHg	11	20.75	8	4.02	6	14.63	28	23.53	7	6.48	3	5.26	136.29	0.001
	Elevated - 140 /90mmHg	18	33.96	109	54.77	10	24.39	35	29.41	34	31.48	21	36.84		
	Seeking medical care due to the high BP- 180mmHg or higher	1	1.89	49	24.62	2	4.88	4	3.36	25	23.15	5	8.77		
	I have not checked it	17	32.08	22	11.06	11	26.83	36	30.25	32	29.63	15	26.32		
	Usually, I have low blood pressure - 100/70mmHg	6	11.32	11	5.53	12	29.27	16	13.45	10	9.26	13	22.81		
What was your cholesterol level?	Don't know	18	33.96	55	28.06	14	34.15	44	36.97	31	28.97	17	29.82	48.34	0.001
	It is high; not taking medicine	4	7.55	48	24.49	11	26.83	14	11.76	17	15.89	12	21.05		
	It was high; taking medicine	20	37.74	81	41.33	10	24.39	36	30.25	55	51.4	21	36.84		
	In norm	11	20.75	12	6.12	6	14.63	25	21.01	4	3.74	7	12.28		

## Discussion

Our study has shown that the metabolic control of diabetes patients during isolation was found to be poor. This is likely due to limited access to medical care. Not measuring glucose can lead to the progression of diabetes complications. Among those who had not measured their glucose levels, 36.82% of patients had multiple micro-macroangiopathies, 31.51% had multiple microangiopathies, 23% had multiple macroangiopathies, 28% had diabetic heart disease, and 40.19% of the same group of patients required emergency care due to a heart attack during isolation. Out of these patients, 31.58% experienced hyperglycemia, and 32% had an arterial hypertensive crisis. The distribution is statistically reliable in both cases. This is most likely explained by the limited medical control during isolation and may also be associated with the socio-economic situation of the region's population.

During the quarantine, 22.25% of respondents did not work, and 15% lost their jobs during the COVID-19 pandemic. The country also does not have continuous glucose monitoring systems, which, according to some studies, has facilitated the management of patients in crisis. The study from Slovenia showed staff contacted established patients with type 1 or type 2 diabetes who performed self-monitoring of blood glucose. A total of 98 patients completed a survey regarding their attitudes towards a home A1c test and telemedicine appointment. The survey showed that patients found the home A1c test and telemedicine appointment convenient and easy to use. Approximately 25% of participants provided qualitative comments, with positive comments focusing on convenience and clear instructions. Satisfaction with the study was high and did not differ by diabetes type, pump or continuous glucose monitor use, or site [22].

Fluctuations in glucose levels also significantly increased the prevalence of complications associated with diabetes mellitus. High numbers of 250 mg% or more and glycemic fluctuations up to 140 to 250 mg% indicated multiple micro- and macroangiopathies at 41.49% vs. 35.11%. The prevalence of cardiovascular disease in diabetes mellitus was high at a glucose level of 140 to 250 mg%, fluctuating at 43.22% vs. 22.8% compared to 250 mg%. Higher glucose levels, on the other hand, significantly increased patients' hospitalization frequency for hyperglycemia by 46.15%. Also, the need for help due to a heart attack or hypertonic crisis was 32.29% vs. 37.16%. The American Diabetes Association and the European Association for the Study of Diabetes updated previous consensus statements on managing high blood glucose levels in adults diagnosed with type 2 diabetes. The recommendations focus on social determinants of health, the healthcare system, physical activity behaviors, weight management, and cardiorenal protection in high-risk diabetes patients [23]. Diabetes proved to be a significant risk factor for severe illness and death from COVID-19. A study of hospitalized COVID-19 patients with diabetes and/or acutely uncontrolled hyperglycemia showed that they had a higher mortality rate and a longer length of stay compared to patients without diabetes or hyperglycemia [24].

Type 2 diabetes is a major comorbidity of COVID-19. A study of 7,337 cases in Hubei Province, China, found that patients with type 2 diabetes had higher mortality rates and required more medical intervention than non-diabetic individuals. Well-controlled blood glucose was correlated with lower mortality rates, while poorly controlled blood glucose was associated with higher mortality rates. Improved glycemic control led to better outcomes in patients with type 2 diabetes and COVID-19 [6].

Glycated hemoglobin levels before and after isolation also significantly altered the prevalence of complications in diabetic patients. Ignorance was the majority of responses, meaning that patients were not aware of or could not participate in the study, almost equally increasing all complications to 60%. For those who had more than 9% glycated hemoglobin before the pandemic, the most common were multiple microangiopathies (18.24%), and in the HbA1c 7% to 8% group, diabetic cardiac diseases were 13.18% and multiple micro- and macroangiopathies were 11.94%. After isolation, when glycated hemoglobin was more than 8%, there was an increase in heart disease by 20.16% and multiple microangiopathies by 18%. With a decrease below 8%, cardiovascular diseases decreased by 17.05%, but macroangiopathy increased by 19.61% and microangiopathy by 24.67%.

There is no reliable data on the difference in glycated hemoglobin levels. Most often, help was required for patients from the "I don't know" group, and in cases with a score of more than 9%, it was likely due to poor metabolic memory in terms of glycemic variability. Regarding blood pressure, multiple micro- and macroangiopathies increased by 27.54% when blood pressure was not measured. With an increase in blood pressure over 140/90mmHg, the growth of all complications was almost the same, and the highest in diabetic cardiac diseases was 43.94%. Cholesterol levels were also unreliable, but "I don't know" was the answer, and it equally increased all the complications. An exciting change was that “I had high cholesterol," took the medication (42.42%), and did not take the medication (15.91%), reflect increased cardiovascular disease associated with diabetes. The quality and dosage of the drug taken can explain this.

In terms of emergency care, the blood pressure distribution is plausible. In the absence of testing, the need for help was high due to hypoglycemia (32.08%), hyperglycemia (26.32%), and heart attacks (29.63%). With an increased blood pressure of 180mmHg or more, the need for help was high due to hypertonic crisis (24.62%) and myocardial infarction (23.15%). An increase in blood pressure of 140/90mmHg increased the risk of crisis (54.77%), infarction (31.48%), hypoglycemia (33.96%), and hyperglycemia (36.84%). Cholesterol levels also significantly increased the need for emergency care during isolation, whereas an unknown level increased the need for help in a hypertonic crisis (28.06%) and the need for help in a heart attack (28.97%). Patients with high cholesterol in group 1, i.e., “I take medicines” for arterial hypertension-crisis (41.33%), had heart attacks (51.4%); group 2, i.e., “I do not take medicines” (24.49%) for arterial hypertension-crisis, required help for hyperglycemia (21.05%), per a study by Ojo et al. This systematic review and meta-analysis, which aimed to analyze the effects of the COVID-19 lockdown on metabolic parameters in patients with type 2 diabetes, identified three areas: glycemic control, lipid parameters, and body mass index. The results showed that the lockdown significantly increased the levels of glycated hemoglobin, fasting glucose, and body mass index. However, the effect of the lockdown on lipid parameters needed to be more consistent [25].

The majority of patients were taking metformin in combination with sulfonylurea and were also using a short- and long-acting (Actrapid-Insulatard) insulin combination or an insulin analog (Apidra-Lantus) combination, which was likely due to the presence of a government diabetes program and a free drug provision program [26]. In Germany, during restrictions, the analysis revealed a significant increase in the number of prescriptions of various drugs between March 2019 and March 2020. In particular, there was an increase of 39% in the prescriptions of angiotensin II antagonists and lipid-lowering drugs. Prescriptions showed a 32% increase in calcium channel blocker (CCB) prescriptions, a 30% increase in beta-blocker prescriptions, a 27% increase in angiotensin-converting enzyme (ACE) inhibitors, vitamin K antagonists (AVAs), and oral antidiabetic drugs, a 24% increase in diuretic prescriptions, and an 18% increase in insulin prescriptions that had indicated decompensation of diabetes [27]. The frequency of use of new-generation anti-glycemic drugs was low. The dipeptidyl peptidase 4 (DPP4) inhibitors were used at 19.3% (n = 139), sodium-glucose cotransporter-2 (SGLT-2) inhibitors at 8.2% (n = 59), and glucagon-like peptide 1 (GLP-1) agonists at 2.6% (n = 19). The American Diabetes Association's diabetes management standards stipulate that in the case of cardiovascular complications, these drugs should be considered first-line therapy after metformin [28].

## Conclusions

Thus, uncontrolled blood glucose levels, as well as extreme fluctuations before and after isolation, significantly raised the prevalence of subsequent complications associated with diabetes and increased the need for medical care in isolation settings. Glucose fluctuations between 140 and 250 mg/dl and above were associated with an increased rate of hospitalization for hyperglycemia and arterial hypertension crises, as well as myocardial infarction. Changes in blood pressure and cholesterol levels were significantly associated with the need for medical care during a pandemic, especially when rates were not monitored. Also, the adoption of new glucose-lowering drugs among diabetic patients was low, which contributed to an increase in the frequency of complications associated with diabetes mellitus.

Thus, it is necessary to educate patients with diabetes that control of metabolic parameters (glucose, glycated hemoglobin, blood pressure, and cholesterol) is of utmost importance, especially in crises and when access to medical care is limited. It would be good if new technologies for continuous glucose monitoring systems appeared in the country. It is crucial to choose hypoglycemic, antihypertensive, and hypolipidemic therapy correctly and also to consider the cardiological advantages of new drugs, which will significantly reduce the frequency of complications of diabetes mellitus.
